# Lectins with Potential for Anti-Cancer Therapy

**DOI:** 10.3390/molecules20033791

**Published:** 2015-02-26

**Authors:** Tammy Yau, Xiuli Dan, Charlene Cheuk Wing Ng, Tzi Bun Ng

**Affiliations:** 1 Department of Neurobiology, Physiology, and Behavior, University of California, Davis, CA 95616, USA; E-Mail: tkyau@ucdavis.edu; 2 School of Biomedical Sciences, Faculty of Medicine, The Chinese University of Hong Kong, Shatin, New Territories, Hong Kong, China; E-Mails: danxiuli@hotmail.com (X.D.); charlene.cw.ng@gmail.com (C.C.W.N.)

**Keywords:** lectins, cancer cells, apoptosis, autophagy

## Abstract

This article reviews lectins of animal and plant origin that induce apoptosis and autophagy of cancer cells and hence possess the potential of being developed into anticancer drugs. Apoptosis-inducing lectins encompass galectins, C-type lectins, annexins, *Haliotis discus discus* lectin, *Polygonatum odoratum* lectin, mistletoe lectin, and concanavalin A, fucose-binding *Dicentrarchus labrax* lectin, and *Strongylocentrotus purpuratus* lectin, *Polygonatum odoratum* lectin, and mistletoe lectin, *Polygonatum odoratum* lectin, autophagy inducing lectins include annexins and *Polygonatum odoratum* lectin.

## 1. Introduction

In a time when biotechnology is rapidly improving, researchers have found a plethora of natural resources to be potential panaceas against cancer. Among such compounds are the lectins family which hold great potential for cancer therapy. Lectins are highly specific proteins that bind to carbohydrates and are found in many plants, animals, and bacteria. Lectins found in animals are most often found to aid in cell interactions, while plant lectins are known to ward off potential predators or pathogens [1]. However, all lectins share the property of involvement in both normal and pathological biological processes and all have varying degrees of interaction with the immune system. Based on their defensive properties and commonly known ability to induce apoptosis in cells, much research has been done to study the effects of plant and animal lectins as a prospective treatment option against cancerous cells. In this review, we will cover just a few of the many plant and animal lectins, including but not limited to *Polygonatum odoratum* lectin, *Haliotis discus discus* lectin, and galectins, that are able to halt growth of tumor cells through apoptotic induction. While a vast number of lectins also show the ability to inhibit cancerous growth through type-II programmed cell death, also known as autophagy, this review will focus primarily on apoptotic methods of inducing cell death in malignant cells in order to limit the amount of information presented. The effectiveness of the specific lectins on particular cancer cell lines and their corresponding pathways and mechanisms will be detailed to provide a basic foundation for prospective researchers. It is imperative to conduct further research on lectins, including clinical trials, to determine which one of these highly specific proteins holds the greatest prospects against the surfeit of malignant cell lines constantly threatening human health and well-being.

While all experiments vary in specifics, there is a general guideline that most researchers follow in order to test the effectiveness and mechanisms of specific lectins on rapidly proliferating cells. Like other biological materials, any lectin to be tested must first be purified from its initial source. This step can vary from study to study but can be done through salting out, dialysis, or various chromatography methods including gel-filtration, ion exchange chromatography, affinity chromatography, and high-pressure liquid chromatography [2]. Gel electrophoresis can then be used to separate out the various proteins and isolate the single protein of interest after chromatographic purification.

The gene encoding a lectin can be inserted into cancer cells using a variety of techniques. The most common method used in laboratories utilizes a virus vector containing the gene coding for a specific lectin. Gel blot hybridizations as well as transfections, a non-viral method of lectin introduction, are also used to insert lectin plasmids into cells to allow production of the lectin [3,4].

Apoptosis can be measured in cells by observing DNA fragmentation, loss of mitochondrial membrane potential, plasma membrane blebbing, shrinking of the cell, condensation of the nucleus, and detachment of the cell. These cellular observations can be determined through flow cytometry and microscopy while specific assays are performed to establish the mechanism of apoptosis induction [5]. Western blot and immunohistochemistry are also techniques employed to further examine mechanisms and gene expression. Lectins can induce apoptosis through different pathways, some being more effective than others in specific cell lines. This can be done by stimulating the production of caspases or other proteins involved in the molecular pathway. Such pathways can lead to down-regulation or up-regulation of certain genes involved in apoptotic suppression or induction, respectively. Certain miRNA act as inhibitors of ribosomal inactivating proteins (RIPs) and can be down-regulated through lectin activity thus allowing RIPs to function properly and inhibit neoplastic growth [6,7].

Research concerning lectins has aided in further discovery of their useful properties for cancer treatment. Not all proteins in the lectin family necessarily need to induce apoptosis to be considered for cancer therapy. Many lectins show potential as biomarkers indicating early detection of malignant growth or as autophagy inducers. Autophagy is a cellular mechanism that involves the catabolic breakdown of cytoplasmic components such as proteins or organelles via lysosomes. Its main function is to remove damaged or infected cells thus contributing to the body’s immune system. Autophagy, like apoptosis, can be induced through multiple pathways and mechanisms depending on the lectin used.

From here on, this review will focus on a few specific animal and plant lectins that have shown apoptotic inducing properties and their mechanisms as outlined in Table 1.

**Table 1:** A list of the main groups of lectins mentioned in this review including the known cancer cell lines affected by each lectin and some of the known mechanisms involved.

Lectin	Cancer Cell Lines Affected	Effector Mechanism(s)
Galectins [8,9,10,11,12]	Epidermal keratinocytes, 1299 lung cells, fibroblast cells, thyroid cells, colon cells, prostate cells	T-cell binding, specific integrin binding, Ca^2+^-calpaincaspase-1 pathway
C-Type Lectins [13,14]	SW1116 colorectal cells	Le glycan recognition, perforin granzyme pathway, TRAIL and FAS ligand binding
Annexins [15,16,17,18,19,20]	Melanoma cells, lung cells	NF-κB signal transduction pathway, Ras-Raf-MAPK pathway, p53 apoptotic pathway
Sialic acid binding *Haliotis discus discus* lectin (HddSBL) [21,22]	Hep3B hepatocellular cells, SW480 colorectal cells, A549 and H1299 lung cancer cell line cells	Bcl-2 down-regulation
*Polygonatum odoratum* lectin (POL) [23,24,25,26]	A549 lung cells, L929 murine fibrosarcoma cells	Akt-mTOR pathway, Fas mediating apoptotic pathway, TNFα enhancement
Mistletoe lectin [7,27,28,29,30,31,32,33]	Hepatocarcinoma cells, breast cancer cells, NALM-6 acute lymphoblastic leukemia cells, glioblastoma cells, hepatomacarcinoma cells, peripheral blood mononuclear cells, A253 epidermoid cells	Wnt signaling, miR-135a & b, NK-mediated cell lysis, interleukin mRNA activation
Concanavalin A (ConA) [34,35,36,37,38,39,40]	A375 and B16 melanoma cells, fibroblast 3T3 cells, colorectal cancer cells	Mitochondrial apoptotic pathway, caspase induction

## 2. Galectin

Galectin is a family of animal β-galactoside-binding proteins that has been found to have members that support cancerous cells by preventing apoptosis, but also some that promote apoptosis in these rapidly dividing cells. Intracellular galectin-3 has many identified pro-cancerous effects such as its interaction with a thyroid-specific transcription factor, TTF-1, subsequently promoting thyroid cell proliferation leading to tumorous growth [8]. Nevertheless, nuclear galectin-3 was discovered to promote apoptosis in human prostate cancer cells [41]. Intracellular galectin-3 is most known as an identifier for both thyroid and prostate cancer. Extracellular galectin-3 has also been found to be both inhibiting as well as promoting apoptosis. Extracellular galectin-3 can decrease T-cell activity by binding to the T-cell receptor complex or induce human T-cells to undergo programmed cell death through CD29 and CD7, two surface glycoproteins, binding which activates an apoptosis signal to the mitochondria [8]. This, therefore, promotes tumor growth as apoptosis of T-cells hinders the immune system.

Suppression of Gal-3 by siRNA or Gal-3 antagonist GCS-100/modified citrus pectin (MCP) promoted cisplatin- triggered apoptosis of PC3 prostate cancer cells which may be mediated by the calcium-dependent protease calpain. Gal-3 inhibition enhances while Gal-3 overexpression inhibits calpain activation. Calpain activation leads to cleavage of androgen receptor into an androgen-independent isoform in prostate cancer cells. Inhibition of calpain with calpain inhibitor and/or siRNA undermined the proapoptotic effect of Gal-3 inhibition, indicating that mechanism for the proapoptotic effect of Gal-3 inhibition may involve calpain activation. Hence the use of a non-toxic anti-Gal-3 agent in conjunction with a toxic chemotherapeutic drug may be a useful therapeutic strategy for chemoresistant prostate cancers [9].

Galectin-1 binding caused T cell surface glycoproteins to redistribute into segregated membrane microdomains on the cell surface. CD45 and CD3 were localized on large islands on apoptotic blebs projecting from the cell surface, which included externalized phosphatidylserine. CD7 and CD43 were localized in small patches away from the membrane blebs, which excluded externalized phosphatidylserine. Receptor segregation was not observed on cells that did not undergo apoptosis following exposure to galectin-1, including mature thymocytes, signifying that receptor redistribution into microdomains is crucial for eliciting apoptosis [42].

Galectin-1 (Gal-1) sensitizes human resting T cells to Fas (CD95)/caspase-8-mediated apoptosis involving a rise in mitochondrial membrane potential and the ceramide pathway. Gal-1 brings about mitochondrial coalescence, budding, fission and an upregulates and/or redistributes fission-associated molecules h-Fis and DRP-1 in resting as well as activated human T cells. This offers a basis for the immunomodulatory action of Gal-1 in experimental cancer models [43].

Galectin-1 triggers apoptosis in specific thymocyte subsets and activated T cells. Galectin-1 exhibits binding to N- and O-glycans on CD7, CD43, and CD45. Galectin-1 signaling in cells expressing low-molecular-weight isoforms of CD45 differs from that in cells expressing a high-molecular-weight isoform of CD45 because the former, but not the latter, necessitates expression of core 2 O-glycans (high-affinity galectin-1 ligands). The findings indicate that the presence of a larger quantity of core 1 O-glycans (low-affinity ligands for galectin-1) can offset the deficiency of core 2 O-glycans. Galectin-1 signaling regulation by α-2,6-sialylation of N-glycans does not only rely on CD45 phosphatase activity. Modulation can be attributed to the relative expression of enzymes that attach sialic acid in an α-2,6- or α-2,3-linkage. The modulation of galectin-1 T cell death by N- and O-glycans takes place through different mechanisms. Thymocytes can be made either susceptible or resistant to galectin-1 by different glycosylation events [44].

Gal-1 demonstrates proapoptotic activity on T-cells. Radiotherapy-induced tumor Gal-1 secretion in mice implanted with Lewis lung carcinoma led to systemic lymphopenia and brought about progression of tumor by intratumoral immunosuppression and augmented angiogenesis. Patients who have received radiotherapy exhibited elevated plasma Gal-1 and reduction in number of T-cells [45].

Galectin-1 (GAL1) is upregulated in a variety of cancers, e.g., in osteosarcoma tissues, and in osteosarcoma patients demonstrates a positive correlation with distant metastasis. GAL1 knockdown inhibited cell proliferation and invasive potential and elicited apoptosis in osteosarcoma cells with attenuated expression of Ki-67, matrix metallopeptidase-9, p-ERK, p38MAPK, and heightened expression of caspase-3. A reduction in tumor size was observed in the MG-63 subcutaneous tumor models after GAL1 treatment compared with the negative control group. Thus GAL1 is a potential target for cancer treatment [46].

β-Galactoside binding protein, a physiological inhibitor of class 1A and class 1B phosphoinositide 3-kinase, elicits apoptosis in aggressive BT474 and SKBR3 breast cancer cells where ErbB2 is overexpressed. The relationship between ERK, akt mRNA, phosphoinositide 3-kinase and cell vulnerability to beta galactoside binding protein challenge was sustained both in MCF10A mammary ductal cells and in non-invasive MCF-7 breast cancer cells compelled to display an aggressive phenotype. β-Galactoside-binding protein has the potential to be safely tested in clinical trials [47].

In addition, galectin-1 overexpression is suspected to be involved in the initial stage of tumorigenesis as it is positively correlated with cell transformation [9]. Cell adhesion depends on interactions between proteins and carbohydrates, and galectin-8 has been found to negatively affect the adhesive ability of human carcinoma 1299 cells and also induce p53-independent cell apoptosis [10]. However, other galectins show no effect on cell adhesion suggesting a unique specific binding to complex cell surface carbohydrates by galectin-8. The capacity of galectin-8 to bind with integrins has been studied and is thought to be the cause of galectin-8’s regulatory ability of cell adhesion and apoptosis. Variation in effectiveness of galectins on cancerous cells depends on cell types as well as concentration. Galectin-1 has been found to be more effective on various carcinomas including epithelial tumors, galectin-7 on thyroid tumors, galectin-8 on colon cancer, and galectin-12 on fibroblast cells [8,9]. While previously mentioned that galectin-1 has pro-cancerous effects, it has been also found to have anti-proliferative properties in epithelial carcinoma cells through binding to the α5β1 integrin. This specific integrin binding stimulates p21 transcription and stabilizes the p27 protein leading to G1 cell cycle arrest and thus inhibits growth [11]. Increased expression of galectin-7 was found in apoptotic human keratinocytes damaged by UVB radiation [12]. It is important to note that many galectins such as galectin-1 and -3 are known to have both anti-cancerous effects as well as pro-cancerous properties therefore warranting further studies in order for effective and correct therapeutically recommendations.

## 3. C-Type Lectins

Lectins such as Dendritic Cell-Specific Intercellular adhesion molecule-3-Grabbing Non-integrin (DC-SIGN), Natural killer-receptors (NK-receptors), and selectins including P-selectin, L-selectin, and E-selectin are all part of the superfamily called C-type lectin. C-type lectins are known to be involved in immune response, cell proliferation, and programmed cell death making them expected targets for research. DC-SIGN is one important C-lectin that can be recognized by glycosylated ICAM-2 which binds to form a DC-SIGN-ICAM-2 complex. This complex begins the maturation of dendritic cells that are capable of generating a specific cytotoxic T lymphocyte-modulated immune response which promotes antitumor activity. Another study also found DC-SIGN can recognize and bind to certain Le glycans expressed in human colorectal carcinoma cells leading to increased immunofunctioning [13]. Tumor death can be triggered by natural killer (NK) cells through the perforin granzyme pathway or death receptors on tumor cells surfaces like TRAIL and Fas ligands [14]. However, NK cells can only be effective cancer treatment options if they can be delivered to the area of the cancerous cells. Unlike the other two C-type lectins previously mentioned, selectins, another group of C-type lectins, are not known to have any apoptotic properties and are more often studied for their role in tumor metastasis through cell adhesion and expression in cancer cells [9]. Nevertheless, they are worth mentioning as they hold great potential for this different method of carcinoma treatment.

## 4. Annexin

Annexins are Ca^2+^-regulated phospholipid-binding proteins that are engaged in regulation of cell growth as well as induction of apoptosis. Researchers have discovered that many animal proteins in the annexin family have apoptotic-inducing properties making them interesting subjects for cancer research. Annexin-1 has been shown to inhibit activation of the NF-κB signal transduction pathway in human cancer cells making it a possible cancer treatment option [15]. The NF-κB signal transduction pathway is often enhanced or constitutively active in cancerous cells and can increase their proliferation or protect them from cell death [16]. As a result, using Annexin-1 as a pathway inhibitor may be efficacious in cancer therapy. Annexin-6 also holds some prospects, acting as a tumor suppressor through negative regulation of the Ras–Raf–mitogen-activated protein kinase (Ras-Raf-MAPK) signaling pathway [15]. The Ras-Raf-MAPK pathway is recognized for its role in cell proliferation, and mutations can create oncogenes associated with the pathway leading to the development of certain cancers [17]. One investigation disclosed that annexin-6 impaired tumor growth in mice and another study showed down-regulation of annexin-6 in metastatic melanoma cells [18,19]. Annexin-7 has been shown to be associated with suppression of prostate cancer cells [20]. Additional research should be done in order to discover more about the mechanisms and pathways involved in restriction of cancer growth by annexins. The regulation of annexin expression in cancer cells such as the increased expression of Annexin-1 in prostate cancer cell lines, esophageal cancer, and hepato-carcinoma also merits further investigation of annexins [9].

## 5. Other Animal Lectins

A wide variety of lectins found in invertebrates act as the first line of defense against pathogens through binding and neutralization or by promoting phagocytosis from other cells [48].

## 6. Marine Animal Lectins

There are a few available studies that have shown isolated marine lectins with the ability to induce apoptosis in cancer cells. However, marine lectins show great potential for anti-cancer treatment due to the fact many of them possess the ability to bind to terminal sugars of glycolipids or glycoproteins, an essential role in marine innate immunity.

### 6.1. Sialic Acid Binding Haliotis Discus Discus Lectin (HddSBL)

Lectins, extracted and purified from *Haliotis discus discus*, also known as disk abalone, are important in the innate immunity process of cells including cell recognition and defense. HddSBL was found to have significant growth obstruction at high enough dosages on Hep3B—a hepatocellular carcinoma cell line, SW480—a colorectal cancer cell line, and A549 and H1299—both lung cancer cell lines [21]. Analysis through cell lysis and western blotting determined that HddSBL caused down-regulation of Bcl-2, an anti-apoptosis factor, but did not activate capases [22]. Capases, also known as cysteine-aspartic proteases or cysteine-dependent aspartate-directed proteases, are important proteins involved in inducing apoptosis in cells.

### 6.2. Fucose-Binding Dicentrarchus Labrax Lectin (DIFBL)

*Dicentrarchus labrax* fucose-binding lectin (DIFBL) is a lectin found in *Dicentrarchus labrax*, more commonly known as the European seabass, and is present in the larvae one month following hatching [49]. In a similar experiment to the one conducted testing the anti-tumor abilities of HddSBL, DIFBL was tested against liver cancer cell lines—Hep3B and BEL-7404, A549 lung cancer cells, and SW480, a colorectal carcinoma cell line. Results showed suppressed *in vitro* proliferation of all the cell lines though effectiveness was dependent on the dosage of DIFBL and length of culture time. The mechanism of apoptosis induction, the cause of proliferation suppression in the cell lines, was found to be through down-regulation of Bcl-2 and XIAP, anti-apoptosis factors, in Hep3B cells which were chosen to be the model studied. Further examination suggested involvement of the PRMT5-E2F-1 pathway in DIFBL induced apoptosis in Hep3B cells despite no up-regulation of caspases [50].

### 6.3. Rhamnose-Binding Strongylocentrotus Purpuratus Lectin (SpRBL)

Rhamnose-binding *Strongylocentrotus purpuratus lectin* (SpRBL) was also found to undergo a mechanism similar to DIFBL to induce apoptosis in Hep3B cells involving the same PRMT5-E2F pathway and likewise down regulating Bcl-2 and XIAP [50]. However, SpRBL was found to be more effective than DIFBL after 72 h of incubation, and while DIFBL has been seen to concentrate in membranous areas and organelles of cells, SpRBL does not show the same localization. Little literature concerning the effects of SpRBL as a cancer therapy option is available, but as it shows consistent results as with other marine lectins, it is an area of lectin research that should be considered for further experimentation.

## 7. Other Plant Lectins

The greatest amount of research on lectin proteins concerns plant lectins due to their widespread occurrence and relatively similar defensive properties. Here we cover the plant lectins that have been found to hold the greatest potential for anti-cancer growth including *Polygonatum odoratum* lectin, mistletoe lectin from a variety of sources, and concanavalin A (Con A). Plant lectins affect both apoptosis and autophagy by modulating representative signalling pathways involved in Bcl-2 family, caspase family, p53, PI3K/Akt, ERK, BNIP3, Ras-Raf and ATG families, in cancer [51].

### 7.1. Polygonatum Odoratum Lectin (POL)

*Polygonatum odoratum* lectin (POL) is a lectin categorized as part of the GNA-related family—a group of lectins all sharing a common three dimensional structure despite having differences in primary amino acid sequence. All GNA-related family lectins specifically bind to the monosaccharide mannose due to a specific amino acid sequence that is present in all such lectins which might be linked to their anti-cancerous properties [23]. POL has been found to induce signs of apoptosis in A549 lung cancer cells without affecting healthy HELF lung cells which did not exhibit signs of membrane blebbing, volume reduction, and DNA fragmentation. The selectivity of apoptosis induction in the malignant A549 cells but not standard HELF cells portends potential tumor suppression. In an experiment, the inhibitory rate was almost 50% after incubating A549 cells for 24 h when a concentration of 23 µg/mL of POL was used and it was determined that apoptosis was induced by means of suppressing a mitochondrial-mediated pathway known as Akt-NF-κb pathway. In addition, autophagy, another form of stopping cancer cell proliferation, was found to be induced by blocking the Akt-mTOR pathway [24]. Another experiment also found POL’s 50% inhibitory rate of L929 murine fibrosarcoma cells to be at a concentration of 25 μg/mL after 24 h of incubation. It was further determined that POL induced apoptosis in the L929 rodent cells through the involvement of a caspase-dependent pathway—consistent with the results found using A549 cells, Fas mediating apoptotic pathway—a death-receptor pathway, and a mitochondrial pathway [25]. Even more promising, POL was shown to enhance the effects of TNFα, a tumor necrosis factor, in the same experiment. POL triggered apoptosis and autophagy in human MCF-7 breast cancer cells by targeting epidermal growth factor receptor-mediated Ras-Raf-MEK-ERK signaling pathway [26].

### 7.2. Mistletoe (Viscum Album) Lectin

Extracts of lectins from mistletoe plant species have been well studied due to their widespread effectiveness on a variety of neoplastic cells, yet it is one of the more controversial lectins when regarding cancer treatment. Many studies show pro-apoptosis effects when certain dosages of mistletoe lectins are given, while other concentrations produce anti-apoptotic consequences [27]. Genes implicated in glioblastoma progression and malignancy including transforming growth factor-β and matrix-metalloproteinases manifested downregulation and tumor growth in glioblastoma xenograft bearing mice was retarded after treatment with the lectin-containing mistletoe extract ISCADOR[28]. Mistletoe lectin extracts are composed primarily of mistletoe lectin I, II, and III though the differences in each one have yet to be studied thoroughly. Research studies have found that lectin purified from the Korean mistletoe, known as *Viscum album* var. coloratum agglutinin (VCA), has positive effects when trials were carried out on human breast cancer cells. Even stronger programmed cell death results were found when the VCA lectin was combined with doxorubicin (DOX), though its clinical uses are limited as DOX is known to have toxic side effects including cardiotoxicity and myelosuppression [29]. Stimulation of proteins inducing apoptosis such as Bax and Puma and inhibition of Bcl-2 was seen when using the combination of VCA and DOX [41]. Korean mistletoe lectin (*Viscum album* L. *coloratum* agglutinin) elicited apoptosis in SK-Hep-1 (p53-positive) and Hep 3B (p53-negative) human hepatocarcinoma cells through p53- and p21-independent pathways, by down-regulation of Bcl-2 and telomerase and up-regulation of Bax functioning upstream of caspase-3 in both cell lines [30].

Chinese mistletoe lectin-1 (CM-1) is an additional lectin that can induce apoptosis in colorectal cancer cells through down-regulation of miR-135a&b expression and up-regulation of expression of the adenomatous polyposis coli (APC) gene leading to reduced activity of Wnt signaling, a gene downstream of APC. Wnt signaling controls β-catenin levels thus affecting gene expression and interference with this signal has been linked to 90% of colorectal cancer cases [7]. Other human cancer cell lines affected by mistletoe lectins include acute lymphoblastic leukemia cells (NALM-6), glioblastoma cells through NK-mediated cell lysis, hepatoma carcinoma cells through a p53- and p21-independent mitochondrial controlled pathway, peripheral blood mononuclear cells via enhanced pro-apoptotic proteins, monocytic tumour cell lines via activating expression of individual interleukin mRNA, and epidermoid carcinoma cells (A253) by inducing dephosphorylation of Akt [31,32]. Recombinant mistletoe lectin increased the survival rate and survival time of severe combined immunodeficient (SCID) mice which had received transplantation of human ovarian cancer cells [33].

### 7.3. Concanavalin A (ConA)

Concanavalin A is a legume lectin that can be extracted from Jack bean seeds. Con A has been found to induce caspase-dependent apoptosis in human melanoma A375 cells. In A375 cells treated with Con A, cytochrome *c* levels were increased which stimulated caspace-9 and caspase-3 levels thus indicating involvement of a mitochondrial apoptotic pathway in Con A-generated apoptosis. This is further supported through findings of mitochondrial transmembrane potential collapse in A375 cells. Also discovered is Con A’s ability to induce autophagy in hepatoma cells through a mitochondrial pathway and glioblastoma cells. Clinical use of Con A is still questionable due to its strong cytotoxic effects that include the induction of hepatitis which would undoubtedly be unsafe for cancer patients. Research findings indicated that a low, non-toxic concentration of Con A inhibited hepatic metastasis of Colon-26 colon cancer cells through an NK-mediated mechanism. *In vivo* murine experiments have also found successful proliferation inhibition using Con A on B16 melanoma cells and fibroblast 3T3 cells [34,35,36,37,38,39].

Con A inhibits the membrane-mediated phosphatidylinositol 3 kinase/Akt/mTOR (mammalian target of rapamycin) pathway and upregulates the MEK/Extracellular signal-regulated kinases (ERK) pathway in HeLa cells resulting in autophagy [40]. Con A elicits autophagy in hepatoma cells through internalization and mitochondrion- mediated pathway which entails a mitochondrial interacting protein designated as Bcl2/E1B-19kDa protein-interacting protein 3 [52].

### 7.4. Soybean (Glycine Max) Lectin

Soybean lectin elicited apoptosis, autophagy, and DNA damage in HeLa cells via the generation of reactive oxygen species. N-acetylcysteine which scavenges scavenger, reactive oxygen species attenuated the action of soybean lectin [53].

### 7.5. Clematis Montana Lectin

Modification of tryptophan and arginine residues and sulfhydryl groups of *Clematis montana* lectin, a mannose-binding lectin resulted in reduction of its anti-proliferative and hemagglutinating activities [54,55].

### 7.6. Sclerotium Rolfsii Lectin

The lectin from the phytopathogenic fungus *Sclerotium rolfsii* strongly inhibited proliferation and induced apoptosis of MCF-7 and ZR-75 human breast cancer cells but only weakly inhibited proliferation of non-tumorigenic MCF-10A and HMEC human breast cells. Botin-labelled *Sclerotium rolfsii* lectin showed little binding to normal human breast tissue but intense binding to cancer issues [56].

## 8. Cellular Targeting of Lectins

The basis for cancer therapy using lectins stems from the ability of these proteins to target multiple cellular components allowing for a wide range of potential cancer treatments. With most lectins, a key component of targeting is cell surface carbohydrates. This is shown in immune defense systems where most lectins follow a general pathway involving carbohydrate recognition of receptors leading to a cascade activation of enzymes such as MBL-associated serine proteases [57]. However, while important, membrane surface carbohydrates are not alone sufficient for lectin-induced apoptosis as shown in a study using BL6-8 melaonma cells and GSIB4 lectins. Even after transfection of the galactosyltransferase (α1,3GT) gene and cell surface α-galactosyl epitopes known to interact with the GSIB4 lectin, the GSIB4 lectin did not induced apoptosis in the BL6-8 cells. Through further testing, the study showed that lectin-induced apoptosis occurs through binding of lectin molecules to a specific receptor, internalization into the cell via endocytosis, and further pathway cascades leading to apoptosis [4]. Oncogenic and tumour suppressive microRNA (miRNA) are also cellular targets of lectins [58]. It has been found that lectins can block carbohydrate-containing receptor EGFR-mediated survival pathways ultimately affecting autophagic hub proteins and miRNAs and inducing autophagy or apoptosis [6]. While wide ranging, Table 2 summarizes a few of the specific cellular targets of the specific lectins discussed in this review involved in apoptosis.

The antiproliferative activity of galectin-1 in various epithelial cancer cell lines necessitates carbohydrate-dependent interaction with the alpha5beta1 integrin. Suppression of the Ras-MEK-ERK cascade by Gal-1 enhances Sp1 transactivation and DNA owing due to diminished threonine phosphorylation of Sp1. Gal-1 stimulates p21 transcription and augments p27 protein stability. Gal-1 mediates accumulation of p27 and p21 suppresses cyclin-dependent kinase 2 activity eventually leading to G(1) cell cycle arrest and growth inhibition [11].

Con A binds to cell membrane glycoproteins, gains entry into the cells and is preferentially located in the mitochondria, leading to alterations in mitochondrial membrane permeability and a pathway of autophagy comprising LC3-II formation, double-layer vesicle, BNIP3 induction, and acidic vesicular organelle formation is triggered. Either 3-MA or siRNA for BNIP3 and LC3, but neither beclin-1 nor ATG 5, partially inhibited the Con A-elicited cell death [59].

**Table 2:** Cellular targets of lectins leading to apoptosis.

Lectin	Cellular Target
Galectins [11,60,61]	Galectin 1: α5β1 integrin
	Galectin 3: oncogenic K-Ras protein
	Galectin 9: antigens presented on T-cell, Ca^2+^ levels, calpain and caspase-1
C-Type Lectins [62]	Myeloid C-type lectin receptors
Annexins [63]	Bax and caspase-3
Sialic acid binding *Haliotis discus discus* lectin (HddSBL) [22]	Bcl-2
*Polygonatum odoratum* lectin (POL) [26]	Bcl-3 and LC3
Mistletoe Lectin [30,64]	Caspase-8, caspase-9, caspase-3, Bcl-2, and telomerase activity
Concanavalin A (ConA) [36,65]	Surface glycoproteins such as mannose sugars, matrix metalloproteinase, cytochrome c, and caspase-3 and -9

## 9. Lectins for Apoptosis-Induced Chemotherapy

With the many natural sources of lectins and the wide supporting evidence for their anti-cancer properties, it seems unwise not to further current research on using lectins for cancer treatment. In this review, we have discussed only a few of the many lectins that researchers have found to have the ability to halt tumor growth through type-I programmed cell death, also known as apoptosis. Cells that have been mutated in a way such that they lose their ability to undergo apoptosis are unable to die and maintain cell homeostasis [66]. These cells are likely to become malignant tumors and will continue to proliferate uncontrollably. Lectin insertion, however, can induce cellular pathways that allow apoptosis to occur and therefore are a viable option for terminating neoplastic growth. Studying the mechanisms by which specific lectins induce apoptosis in cancer cells is important for understanding their effectiveness on different cancer cell lines and should be further examined. Further research on how lectins can be implemented as a drug to patients is also just as important for successful treatment as clinical trials are a necessary step in developing lectins as an anti-cancer drug. Apoptotic means are not the only way to inhibit harmful proliferation. Type-II programmed cell death, more commonly referred to as autophagy, has been documented as a mechanism by which certain lectins prevent further cancerous growth as well as through ribosomal deactivation. This review not only aims to support the necessity for further investigation of specific lectin potential, but to also aid in starting the foundation for studies on lectin effects on malignant cell growth.

Understanding the process and mechanisms by which lectins affect cancerous cell growth is impertinent if we are to use lectins in future clinical cancer therapy. It is important to realize the discrepancies between animal model experiments and the results found in humans when considering the use of lectins for cancer treatment. Lectins are known to have some toxic effects at high concentrations creating some complications when regarding their use for therapy. Mistletoe lectins are a standard example of a potential anti-tumor compound that produces debatable effects when examined in cancer patient prospective studies. While research studies *in vivo* and *in vitro* involving mistletoe lectins both show anti-tumor effects, clinical studies have yet to support such findings. Positive trends have been seen in studies using mistletoe lectins on breast cancer patients, but literature reviews of earlier studies have shown little conclusive evidence for the benefits of mistletoe lectins on survival or quality of life of patients but data from recent years tend to suggest a benefit [67,68,69,70,71,72,73,74,75,76,77]. However, this does not mean we should refute all experimental lectin trials as many such prospective studies lacked proper methodological practices and were possibly subjected to potential bias. Furthermore, as previously stated, all lectins are known to have varying degrees of effectiveness based on dosage or specific type of lectin extracted which must be taken into consideration when reviewing the literature of human clinical trials. Investigations using *in vivo* lectin experiments may provide a better indication on how a specific lectin may react in a clinical setting and what cytotoxic effects the lectin may hold. Con A is a case in which the lectin has anti-neoplastic effects but can also have toxic effects on cells [78].

Besides being vital areas for drug testing, lectins also hold potential for use as cancer markers and predictors. Certain single nucleotide polymorphisms found in the genes coding for C-type lectins have been found to be associated with increased risk of developing colorectal cancer and its severity if established though further studies are warranted [79]. Many galectins including galectin-1 and galectin-3 have also shown a correlation with tumor development and can be used as markers of potential cancerous growth [80]. Both galectin-1 and galectin-3 interact with oncogenic Ras, a proto-oncogene known to be mutated and constantly expressed in tumor cells rendering these two galectins to be favorable targets for therapy [81]. Thus, directing research at such lectins for cancer treatment can be potentially significant and merits further research not only to determine the use of lectins for apoptotic induction, but also to predict and prevent malignant cell growth.

## 10. Autophagy: Another Possibility for Lectin-Mediated Cancer Therapy

Autophagy is an important component in lectin research as a mechanism for inhibiting neoplastic growth. Though this review is concerned mainly the mechanisms of apoptosis induction, it is important to consider the possibilities autophagy presents in lectin cancer therapy. Autophagy is the process by which a cell will destroy its organelles through the use of autophagosomes and lysosomes. While there are two main types of autophagy—microautophagy and macroautophagy—the general term autophagy is commonly used to refer to the process of macroautophagy. In microautophagy, cytoplasm is engulfed directly at the surface of lysosomes, whereas macroautophagy creates autophagosomes by isolating part of the cytoplasm to create a separate vesicle. This autophagosome then fuses with a lysosome for degradation. Autophagy is not only associated with cancer and has been shown to play an important role in anti-aging, cell development, and antigen presentation thus linking autophagy with immune response [82,83,84,85,86].

Studies have shown autophagy defects are linked to tumorous growth through various genes. Beclin 1, a gene in mammals similar to the Atg 6 gene in yeast, is a part of a type-III phosphatidylinositol 3-kinase complex that is necessary for autophagic vesicle formation for tumor suppression. DNA sequencing of human breast and ovarian cancer has shown that Beclin 1 is often lost leading to neoplasm [87]. Tumor suppressor genes, p53 and PTEN, both stimulate autophagy and are common targets for cancer therapy. Autophagic mechanism damage leads to genome instability which often further proceeds to the uncontrolled and malignant cell growth of tumors. In some cancer cell lines that are apoptosis-resistant or defective, autophagy-induced cell death can be stimulated through chemotherapy treatments such as arsenic trioxide, resveratrol, and tamoxifen [88].

In reference to specifically discussed lectins in this review, autophagy can play a key role in their success in inducing cancerous cell death. Con A can bind to mannose glycoproteins and inhibit growth and is suspected to take on an autophagic pathway in heptatoma ML-1 cells [37]. Formation of LC3-II, an autophagy marker, and double-layer vesicles and induction of BNIP3, a protein associated with autophagy, support such conclusions [59,89]. The animal lectin, Annexin-5, has been shown to induce autophagy as well as inhibit endocytosis [90]. Though not discussed in this review, another lectin, 9*Polygonatum cyrtonema* lectin (PCL), was found to induce both autophagy and apoptosis through a mitochondria-mediated ROS–p38–p53 pathway in human melanoma A375 cells [91].

**Figure 1:**
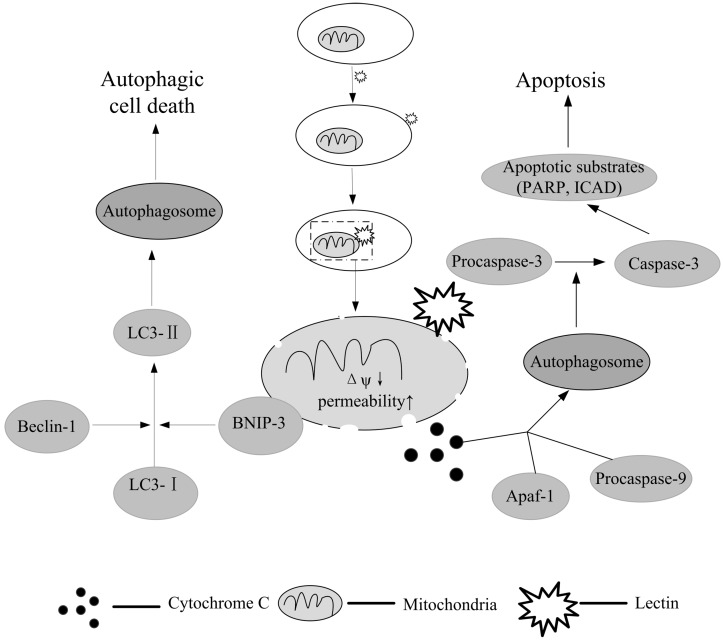
Mechanisms of apoptosis and autophagy induced in tumor cells by lectins.

Another approach involving the use of autophagy in cancer treatment involves the connection between autophagy and apoptosis. Atg5, a specific autophagic protein, has been found to active an apoptotic program when cleaved [92]. Thus the study of autophagic mechanisms may be instrumental in determining successful mechanisms of neoplastic cell death. Figure 1 presents the mechanisms of apoptosis and autophagy induced by lectins in cancer cells.

## 11. Conclusions

In summary, the use of lectins obtained from natural plant and animal sources shows great promise and potential for use in future cancer therapy. Current research has found strong anti-cancer effects of many lectins as shown in this article. Both apoptosis and autophagy are important factors that influence the success of lectin chemotherapy. Additional research should be funded in order to better understand the mechanisms of lectins in both apoptosis and autophagy and aid in the transition from research to clinical application. *In vitro* laboratory and *in vivo* animal studies show promising results, but it is clear that clinical studies are necessary to advance cancer research in utilizing lectins for chemotherapeutic treatment. Mistletoe extract containing lectin has been used clinically at low doses in the treatment of different cancers without serious side effects and the action seems to be beneficial in some cases [93,94,95,96].
